# Supplementary material for: Bile acids-mediated intracellular cholesterol transport promotes intestinal cholesterol absorption and NPC1L1 recycling
Source: Nat Commun. 2023 Oct 13;14:6469. doi: 10.1038/s41467-023-42179-5 (PMC10575946; doi:10.1038/s41467-023-42179-5)
Supplement: Supplementary file 1 — Supplementary Information [file 41467_2023_42179_MOESM1_ESM.pdf]

Supplementary information for

Bile acids-mediated intracellular cholesterol transport promotes intestinal cholesterol absorption and NPC1L1 recycling

Authors and Affiliations:

Jian Xiao[1,#], Le-Wei Dong[1,#], Shuai Liu[1,2], Fan-Hua Meng[1,2,3], Chang Xie[1], Xiao-Yi Lu[1], Weiping J. Zhang[4], Jie Luo[1], Bao-Liang Song[1,*]

[1]College of Life Sciences, Taikang Center for Life and Medical Sciences, Taikang Medical School, Hubei Key Laboratory of Cell Homeostasis, Wuhan University, Wuhan, China

[2]Heart Center, First Affiliated Hospital of Xinjiang Medical University, Urumqi 830054, Xinjiang, China

[3]Affiliated Hospital of Jining Medical College, Jining 272007, Shandong, China

[4]Department of Pathophysiology, Naval Medical University, Shanghai, China

[#]These authors contribute equally to this work.
[*]To whom correspondence should be addressed: E-mail: blsong@whu.edu.cn.

This PDF file includes:

Figs. S1 to S11
Table S1 to S2

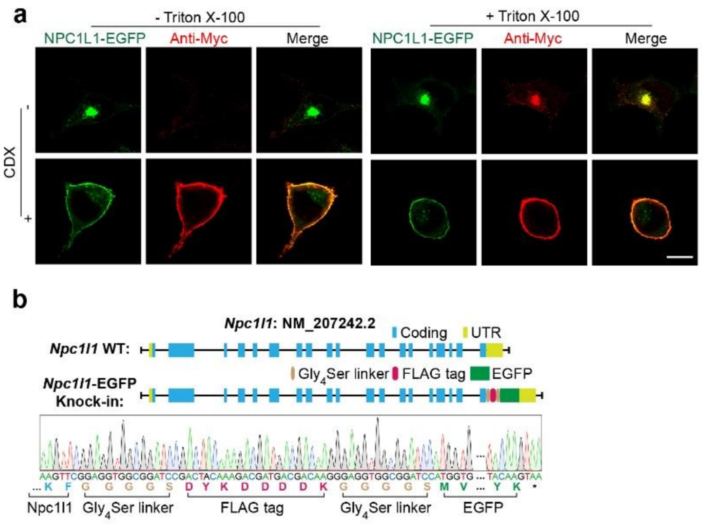

Supplemental Figure 1. Schematic model of *Npc1l1*-EGFP knock-in mouse and validation of NPC1L1-3×Myc-EGFP topology.

a The CRL1601/NPC1L1-3×Myc-EGFP cells were treated with 1.5% CDX for 1 h and stained with the anti-Myc antibody with or without membrane permeabilization induced by Triton X-100 (n=2). Scale bar, 10 µm. **b** The schematic model of *Npc1l1*-EGFP knock-in mouse. The DNA sequence encoding a tandem tag $(Gly)_4$Ser-FLAG-$(Gly)_4$Ser-EGFP was inserted before the stop codon of mouse *Npc1l1*. WT: wild-type; UTR: untranslated region.

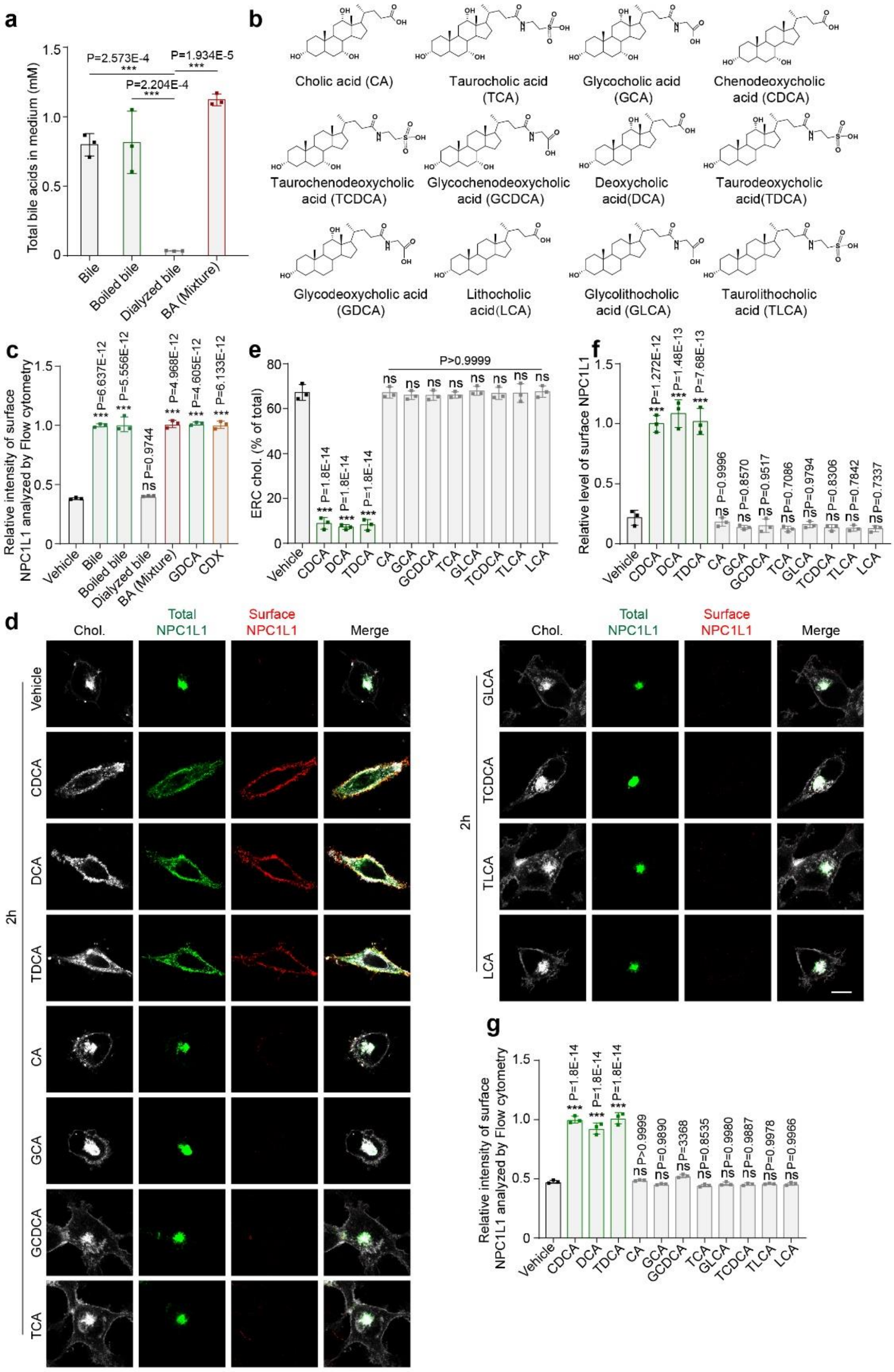

Supplemental Figure 2. The effects of major human bile acids on promoting cholesterol egress and NPC1L1 translocation.

a The concentrations of total bile acids in the medium of cells treated with isochoric bile, boiled bile, dialyzed bile or BA mixture. One-way ANOVA with Tukey *post hoc* test, *** P<0.001; ns, no significance. **b** The molecular structure of 12 major human bile acids. **c** CRL1601/NPC1L1-3×Myc-EGFP cells with 2-h treatment of indicated bile, bile acids or CDX were resuspended by 2 mmol/L EDTA and stained with an

anti-Myc antibody and then secondary antibody without membrane permeabilization. Cells were fixed and analyzed by flow cytometry. The average intensity of surface NPC1L1 in bile-treated cells was defined as 1. Values were presented as mean ± SD (n=3 independent trials, 2000 cells/trial). **d** CRL1601/NPC1L1-3×Myc-EGFP cells were treated with the indicated bile acids for 2 h. Scale bar, 10 µm. **e-f** The percentage of ERC cholesterol (**e**) and the relative intensity of surface NPC1L (**f**) in **d** were quantified. The average intensity of surface NPC1L1 in CDCA-treated cells was defined as 1. Values were presented as mean ± SD (n=3 independent trials, 100 cells/trial). One-way ANOVA with Tukey *post hoc* test, *** P<0.001; ns, no significance. **g** The surface NPC1L1 of CRL1601/NPC1L1-3×Myc-EGFP cells with 2-h indicated bile acid treatment were analyzed by flow cytometry. The average intensity of surface NPC1L1 in CDCA-treated cells was defined as 1. Values were presented as mean ± SD (n=3 independent trials, 2000 cells/trial). Chol.: Cholesterol. Source data are provided as a Source Data file.

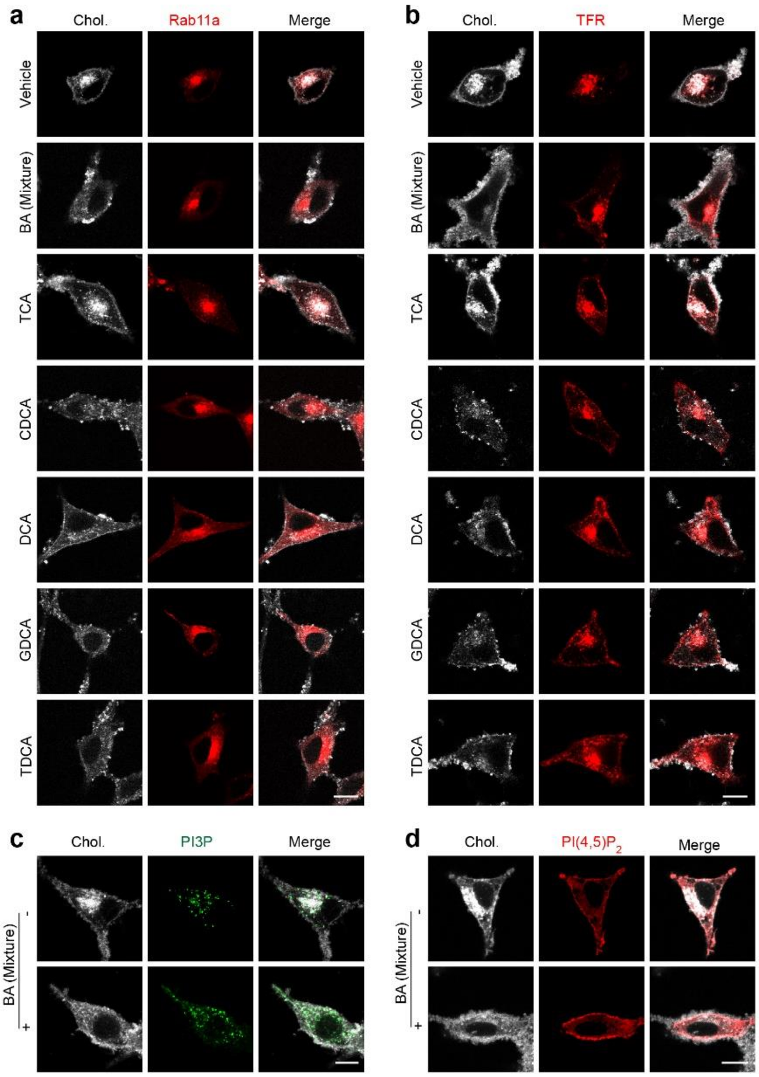

Supplemental Figure 3. Bile acids transport cholesterol away without affecting ERC morphology and phosphoinositides distribution

a-b CRL1601 cells were transfected with pCMV-mCherry-Rab11a (**a**) or pCMV-TFR-mCherry (**b**) for 48 h. Cells were treated with indicated bile acids for 2 h and then fixed by 4% PFA, followed by filipin staining. **c-d** CRL1601 cells were transfected with CMV-EGFP-$FYVE_{SARA}$ (**c**) or CMV-PH-PLCδ-mCherry (**d**) to label PI3P or PI(4,5)P_2, respectively. Then cells were treated with BA mixture for 2 h and fixed by 4% PFA, followed by filipin staining (n=2). Chol: Cholesterol.

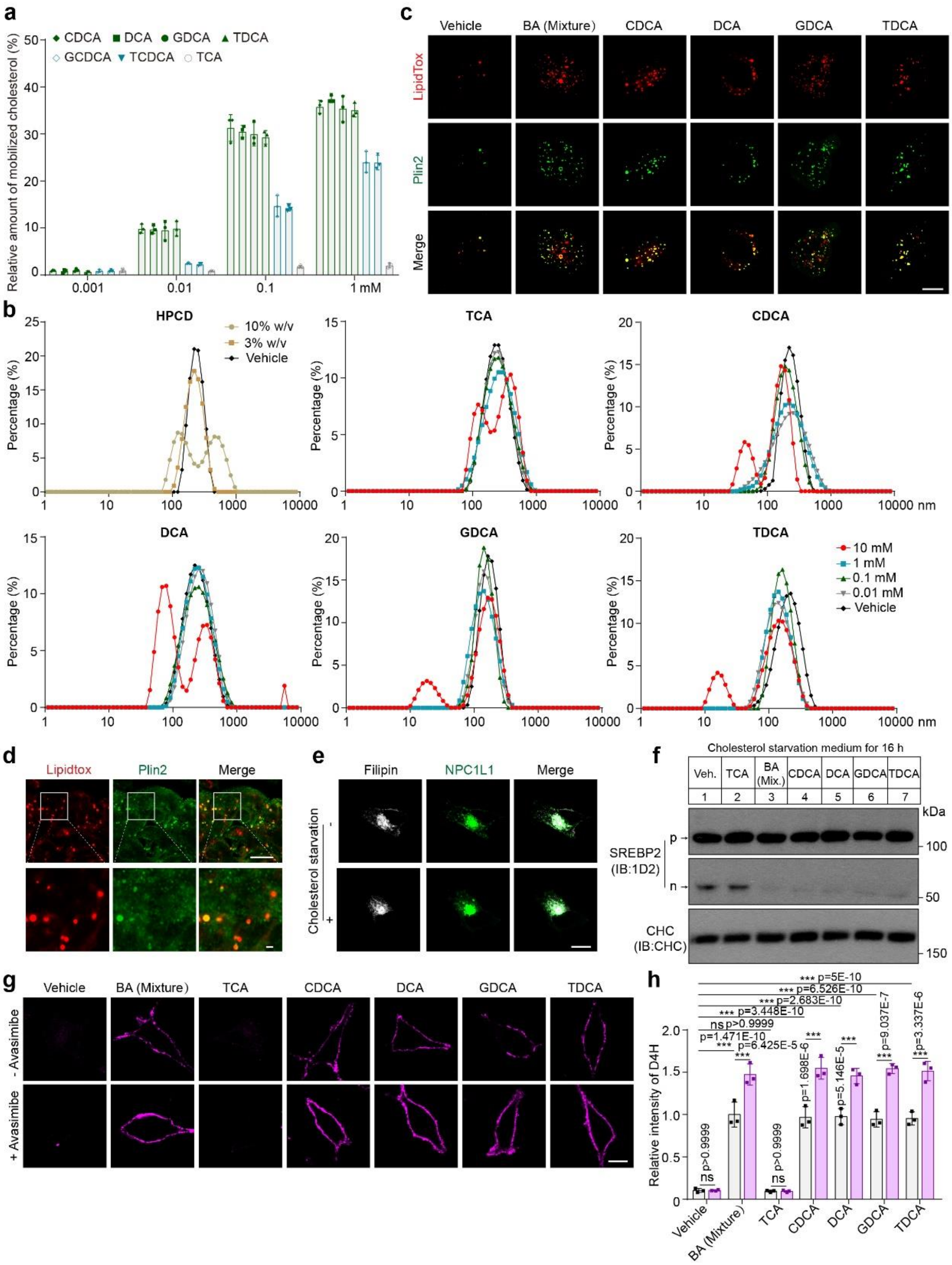

Supplemental Figure 4. Dihydroxy bile acids convey cholesterol *in vitro* at submicellar concentrations and four of them (CDCA, DCA, GDCA and TDCA) can transport cholesterol to ER and PM in cells.

a Percentages of mobilized cholesterol from the *in vitro* cholesterol mobilization assays in response to various bile acids at indicated concentrations. Values were presented as mean ± SD (n=3 independent trials). **b** The diameter distribution of liposomes with the 2-h treatment of HPCD or bile acids at indicated concentrations. The percentage was defined as the ratio of liposomes at a specific size to the total. **c**

CRL1601 cells were transfected with Plin2-3×FLAG (green) for 48 h, treated with indicated bile acids and stained with anti-FLAG antibody, followed by LipidTox staining (n=2). Scale bar, 10 μm. **d** LipidTox and endogenous Plin2 staining in frozen intestinal sections of in vitro cultured intestines from neonatal C57BL/6J male mice. The small intestine section from the neonatal mouse was incubated with 10 μg/mL cholesterol and BA mixture for 2 h. Then section was fixed, stained with the anti-Plin2 antibody, followed by LipidTox staining (n=2). Scale bar, 10 μm (main); 1 μm (inset). **e** CRL1601/NPC1L1-3xMyc-EGFP cells were incubated in DMEM with 10% FBS (-cholesterol starvation) or cholesterol starvation medium (+cholesterol starvation) for 16 h. Cells were fixed and stained with filipin (n=3). Scale bar, 10 μm. **f** CRL1601/NPC1L1-3xMyc-EGFP cells were incubated with cholesterol starvation medium for 16 h and then incubated with indicated bile acids for 2 h prior to harvest (n=2). p, precursor of SREBP2; n, nuclear of SREBP2. **g** CRL1601/NPC1L1-3xMyc-EGFP cells were treated with indicated bile acids in the absence or presence of avasimibe. Cells were stained with recombinant mcherry-D4H prior to fixation. Scale bar, 10 μm. **h** The relative intensity of D4H in **g** was quantified and the average intensity of BA mixture-treated cells in the non-avasimibe group was defined as 1. Values were presented as mean ± SD (n=3 independent trials, 100 cells/trial). Two-way ANOVA with Tukey post hoc test, *** $P<0.001$; ns, no significance. Source data are provided as a Source Data file.

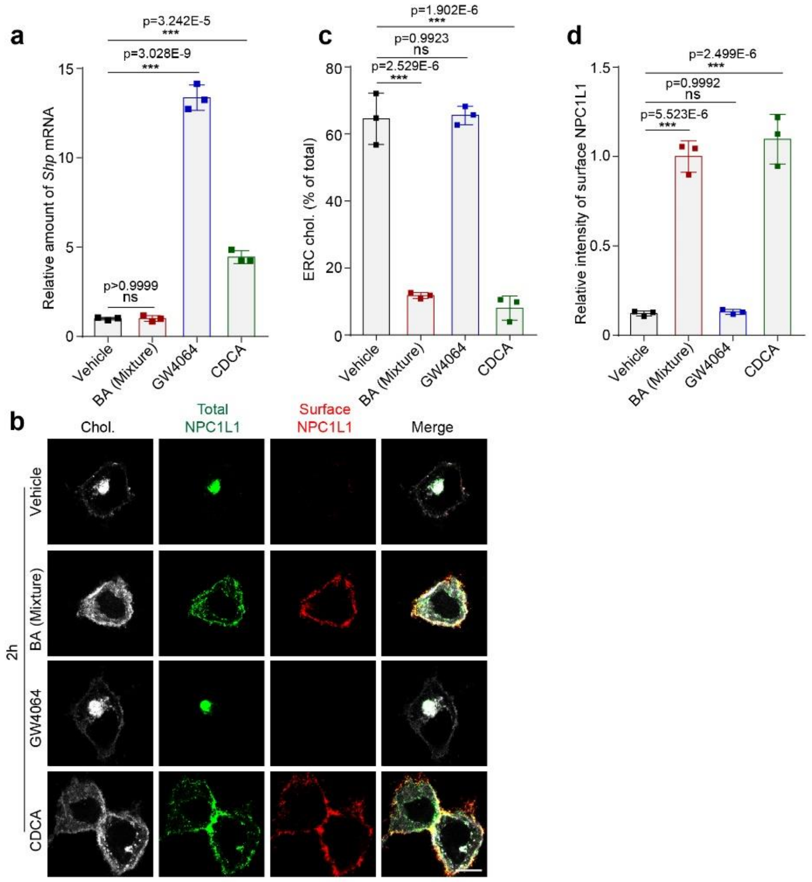

Supplemental Figure 5. CDCA and DCA transport ERC cholesterol to ER and PM via its cholesterol mobilizing ability without involving the FXR pathway.

a The relative mRNA level of FXR target gene *Shp* in CRL1601 cells treated with BA mixture, GW4064 and CDCA for 2 h. (n=3 independent trials). One-way ANOVA with Tukey *post hoc* test, ***P<0.001; ns, no significance. **b** Representative confocal images of CRL1601/NPC1L1-3×Myc-EGFP cells treated with BA mixture, FXR agonist GW4064 or CDCA for 2 h. Scale bar, 10 µm. **c-d** Percentages of ERC cholesterol (**c**) and relative intensity of surface NPC1L1 (**d**) in **b** were quantified. The average intensity of surface NPC1L1 in BA mixture-treated cells was defined as 1. Values were presented as mean ± SD (n=3 independent trials, 100 cells/trial). One-way ANOVA with Tukey *post hoc* test, *** P<0.001; ns, no significance. Chol: Cholesterol. Source data are provided as a Source Data file.

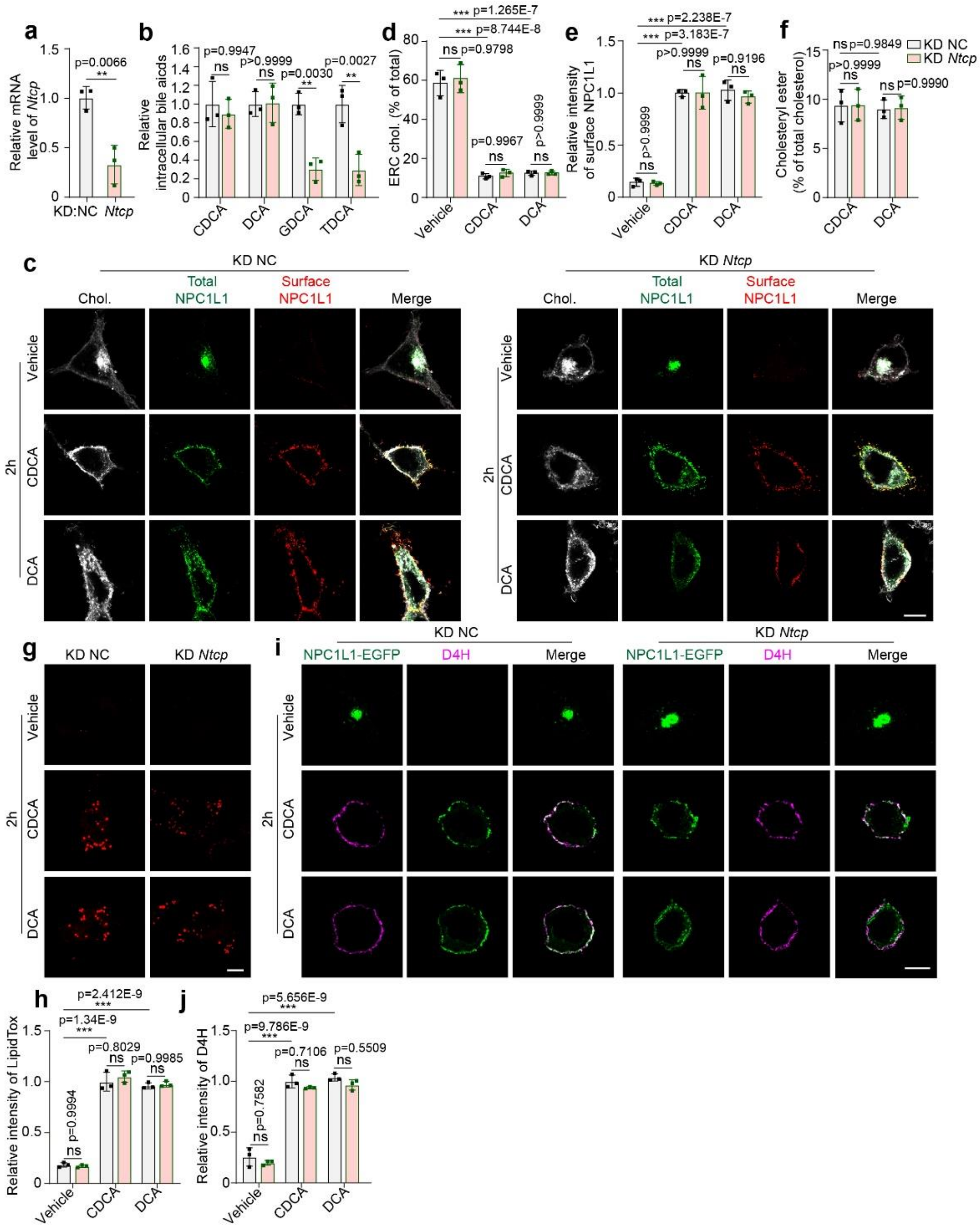

Supplemental Figure 6. Ablation of NTCP has little effect on CDCA or DCA mediated cholesterol transport.

a Knockdown efficiency of *Ntcp* in CRL1601 cells. Values are presented as mean ± SD (n=3). Unpaired two-tailed Student's t-test. **P < 0.01. **b** The relative amount of intracellular CDCA, DCA, GDCA or TDCA in control or *Ntcp* knockdown CRL1601/NPC1L1-3×Myc-EGFP cells with indicated treatment. The average intracellular bile acids of each control groups are defined as 1, respectively. Values are presented as mean ± SD (n=3). Unpaired two-tailed Student's t-test. **P < 0.01; ns, no significance. **c** Representative confocal images of control or *Ntcp* knockdown CRL1601/NPC1L1-3×Myc-EGFP cells treated with indicated treatment. Scale bar, 10 µm. **d-e** Percentages of ERC cholesterol (**d**) and relative intensity of surface NPC1L1 (**e**) in **c** were quantified. The average intensity of surface NPC1L1 in CDCA-treated

control cells was defined as 1. Values were presented as mean ± SD (n=3, 100 cells/trial). Two-way ANOVA with Tukey *post hoc* test, ** P<0.01; *** P<0.001; ns, no significance. **f** Percentages of cholesteryl esters relative to total cholesterol in control or *Ntcp* knockdown CRL1601/NPC1L1-3×Myc-EGFP cells with indicated treatment. Values are presented as mean ± SD (n=3). Two-way ANOVA with Tukey *post hoc* test, ns, no significance. **g** Representative LipidTox staining of control or *Ntcp* knockdown CRL1601/NPC1L1-3×Myc-EGFP cells with indicated treatment. Scale bar, 10 µm. **h** Relative intensity of LipidTox in **g** was quantified. The average intensity of CDCA-treated control cells was defined as 1. Values were presented as mean ± SD (n=3, 100 cells/trial). Two-way ANOVA with Tukey *post hoc* test, *** P<0.001; ns, no significance. **i** D4H staining was performed in control or *Ntcp* knockdown CRL1601/NPC1L1-3×Myc-EGFP cells with indicated treatment. Scale bar, 10 µm. **j** The relative intensity of D4H in **i** was quantified. Values were presented as mean ± SD (n=3, 100 cells/trial). The average intensity of D4H in CDCA-treated cells was defined as 1. Two-way ANOVA with Tukey *post hoc* test, *** P<0.001; ns, no significance. Source data are provided as a Source Data file.

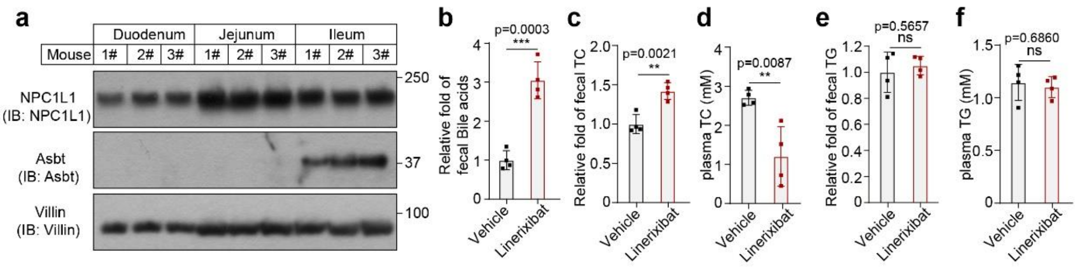

Supplemental Figure 7. Intestinal bile acids transporter ASBT is required for cholesterol absorption

a The protein expression levels of NPC1L1 and Asbt in the duodenum, jejunum and ileum. Three male C57BL/6J mice (nine-week-old) were subjected to the western blot analysis (n=3 mice). **b, c and e** The relative amount of fecal bile acids (**b**), total cholesterol (TC) (**c**) and total triglycerides (TG) (**e**) in male mice (nine-week-old) receiving oral gavage of vehicle or linerixibat daily for 3 days. The mean value of the vehicle-treated group was defined as 1. Values were presented as mean ± SD (n=4 mice per group). **d and f** The total cholesterol (**d**) and total glycerides (**f**) in the plasma of male mice (nine-week-old) receiving oral gavage of vehicle or linerixibat daily for 3 days. Values were presented as mean ± SD (n=4 mice per group). Source data are provided as a Source Data file.

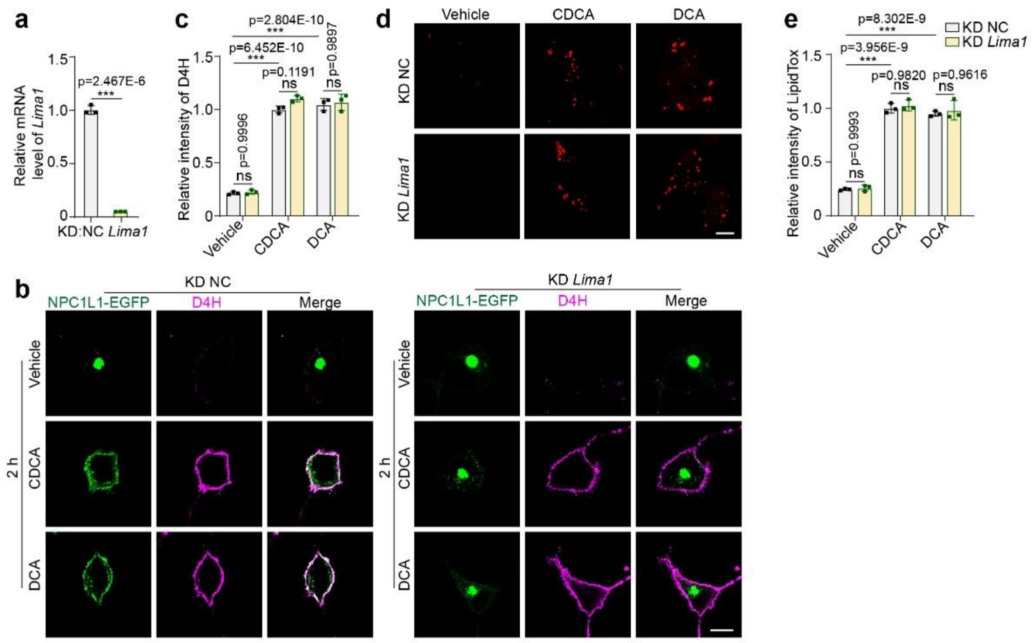

Supplemental Figure 8. Silencing of *LIMA1* does not affect CDCA- or DCA-mediated egress of ERC cholesterol.

a Knockdown efficiency of *Lima1* in CRL1601 cells. Values are presented as mean ± SD (n=3 independent trials). Unpaired two-tailed Student's t-test. *** P<0.001. **b** D4H staining prior to fixation was performed in control or *Lima1* knockdown CRL1601/NPC1L1-3×Myc-EGFP cells treated with vehicle, CDCA or DCA for 2 h. Scale bar: 10 µm. **c** The relative intensity of D4H in **b** was quantified. The average intensity of D4H in CDCA-treated cells was defined as 1. Values were presented as mean ± SD (n=3 independent trials, 100 cells/trial). Two-way ANOVA with Tukey *post hoc* test, *** P<0.001; ns, no significance. **d** Representative LipidTox staining of control or *Lima1* knockdown CRL1601/NPC1L1-3×Myc-EGFP cells after a 2-h incubation of vehicle, CDCA or DCA. Scale bar, 10 µm. **e** Relative intensity of LipidTox in **d** was quantified. The average intensity of CDCA-treated control cells was defined as 1. Values were presented as mean ± SD (n=3 independent trials, 100 cells/trial). Two-way ANOVA with Tukey *post hoc* test, *** P<0.001; ns, no significance. NC: Negative control. Source data are provided as a Source Data file.

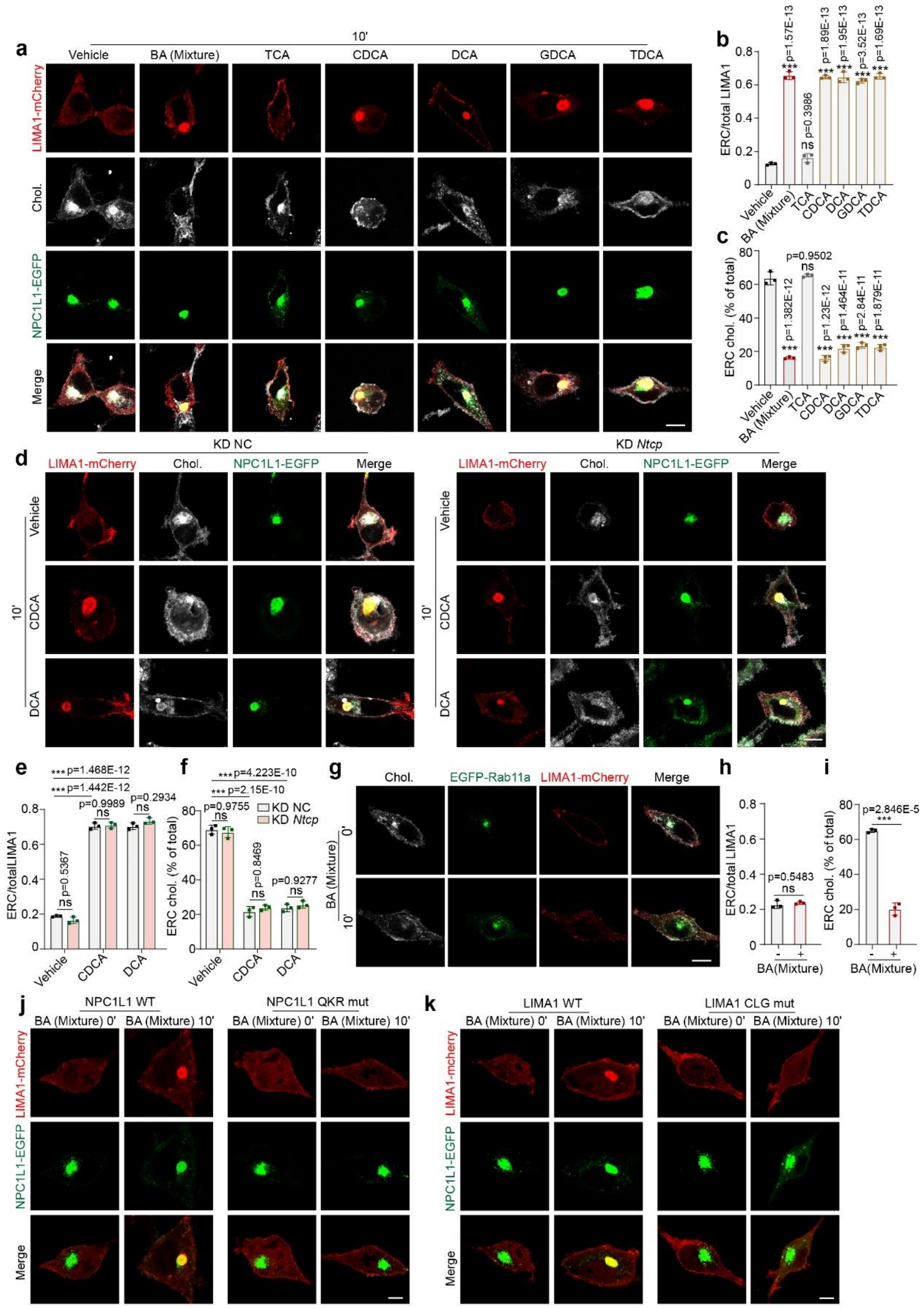

Supplemental Figure 9. Depleting cholesterol by bile acids redistributes LIMA1 to NPC1L1-positive compartment.

a Representative confocal images of CRL1601/NPC1L1-3×Myc-EGFP cells overexpressing LIMA1-mCherry treated with indicated bile acids for 10 min prior to fixation. Cells were fixed and stained with filipin to indicate cholesterol. Scale bar, 10 µm. **b-c** The relative amounts of ERC resident LIMA1 (**b**) and cholesterol (**c**) in **a**

were quantified. Values were presented as mean ± SD (n=3 independent trials, 100 cells/trial). One-way ANOVA with Tukey *post hoc* test, *** P<0.001; ns, no significance. **d** Control or *Ntcp* knockdown CRL1601/NPC1L1-3×Myc-EGFP cells were transfected with pCMV-LIMA1-mCherry and pre-treated with a 10-min incubation of vehicle, CDCA or DCA prior to fixation. Scale bar: 10 μm. **e-f** The relative amounts of ERC resident LIMA1 (**e**) and cholesterol (**f**) in **d** were quantified. Values were presented as mean ± SD (n=3 independent trials, 100 cells/trial). Two-way ANOVA with Tukey *post hoc* test, *** P<0.001; ns, no significance. **g** Representative confocal images of CRL1601 cells co-transfected with pCMV-EGFP-Rab11a and pCMV-LIMA1-mCherry, followed by a 10-min incubation of BA mixture before fixation. Scale bar, 10 μm. **h-i** The relative amounts of ERC resident LIMA1 (**h**) and cholesterol (**i**) in **g** were quantified. Values were presented as mean ± SD (n=3 independent trials, 100 cells/trial). Unpaired Student's *t*-test, *** P<0.001; ns, no significance. **j** CRL1601 cells were co-transfected with pCMV-LIMA1-mCherry and pCMV-NPC1L1-3×Myc-EGFP or Q_{1277}KR→AAA variant (QKR mut) for 48 h, followed by 10-min treatment of bile acids mixture. Scale bar: 10 μm. **k** CRL1601 cells were co-transfected with pCMV-NPC1L1-3×Myc-EGFP and pCMV-LIMA1-mCherry or its C_{164}LG→AAA variant (CLG mut) for 48 h, followed by 10-min treatment of bile acids mixture. Scale bar: 10 μm. Source data are provided as a Source Data file.

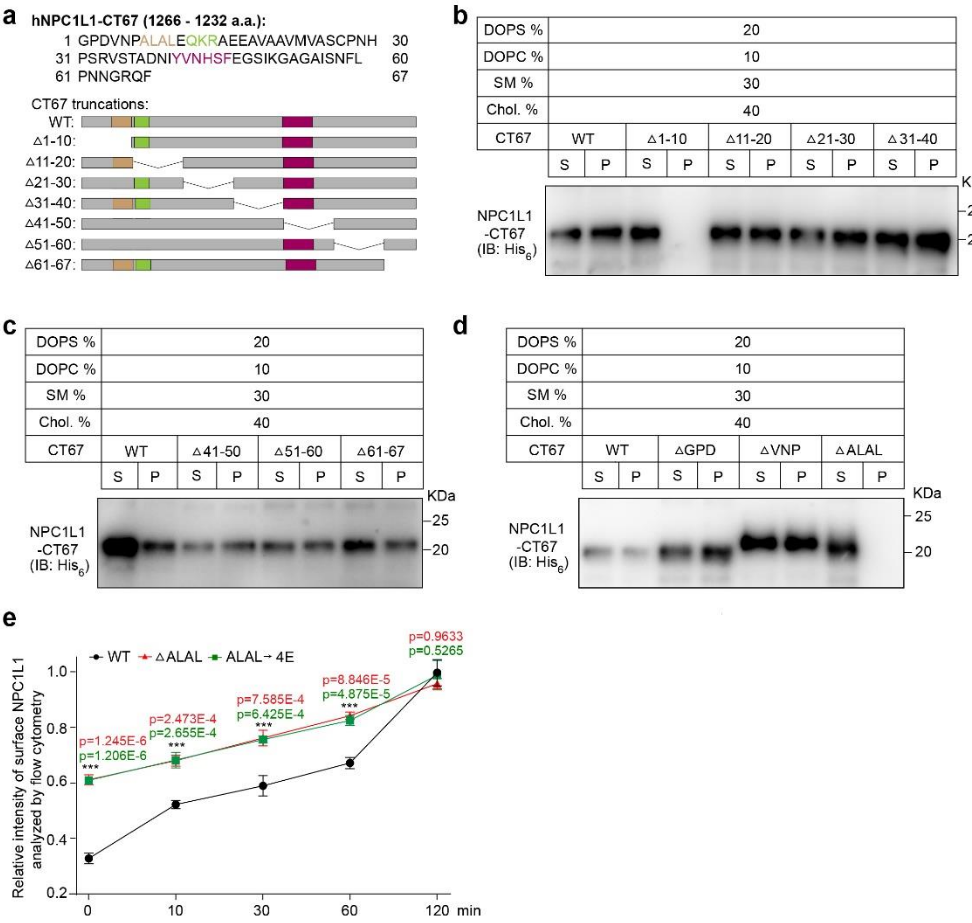

Supplemental Figure 10. The region (1272-1275 a.a.) of NPC1L1 is required for its interaction with cholesterol-liposome.

a Schematic model of NPC1L1-CT67 and its truncations. a.a.: amino acids. **b-d** Liposome co-sedimentation assay using indicated NPC1L1-CT67 truncations (n=2). Interaction of NPC1L1-CT67 truncations (WT, Δ1-10, Δ11-20, Δ21-30 and Δ31-40) and liposomes containing cholesterol were analyzed in **b**. Co-sedimentation assay of NPC1L1-CT67 truncations (WT, Δ41-50, Δ51-60 and Δ61-67) and liposomes were performed in **c**. Further shortened truncations (ΔGPD, ΔVNP, ΔALAL) of NPC1L1-CT67 Δ1-10 amino acids were applied to liposome co-sedimentation assay in **d**. **e** The relative intensity of surface NPC1L1 of CRL1601 cells stably expressing NPC1L1-3×Myc-EGFP, its A_{1272}LAL deletion (ΔA_{1272}LAL) or mutation (A_{1272}LAL→4E) variant following BA mixture treatment over time was analyzed by flow cytometry. The average intensity of cells overexpressing WT 3×Myc-EGFP with 120 min treatment of BA mixture was defined as 1. Values were presented as mean ± SD (n=3 independent trials, 2000 cells/trial). One-way ANOVA with Tukey *post hoc* test. WT: Wild-type. Source data are provided as a Source Data file.

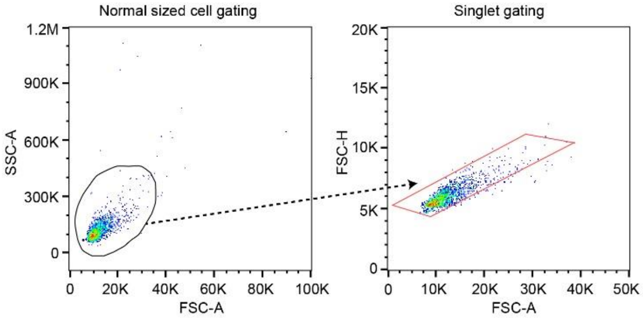

Supplemental Figure 11. Representative gating strategy of surface NPC1L1 quantifications.

The normal-sized stable cells (CRL1601/NPC1L1-3×Myc-EGFP cells, CRL1601/NPC1L1-3×Myc-EGFP-ΔA1272LAL or CRL1601/NPC1L1-3×Myc-EGFP-A1272LAL→4E) were gated using forward scatter and side scatter. Then single cells from the stable cells were gated by forward scatter height and area, and used for measurement of fluorescence intensity.

Supplementary Tables

Supplementary Table 1. Sequences of siRNA and qPCR primers in this study.

siRNAs:	
si-Lima1	5'-AAGCAUCAUCCUGGUGUUAAU-3'
si-Ntcp	5'-GGAAAUAGGUGCAGGUCAU-3'
si-scramble	5'-UUCUCCGAACGUGUCACGU-3'
qPCR Primers:	
Rat *Shp*	F: 5'-CACTATCCTCTTCAACCCAGATGT-3'<br>R: 5'-GGCTCCAGGACTTCACACAA-3'
Rat *Lima1*	F: 5'-TGTCCGTGGAGGAGCAGATA-3'<br>R: 5'-TCCCACGTAAAAGACCCGTG-3'
Rat *Ntcp*	F: 5'-AGGTGCACAACGTATCAGCC-3'<br>R: 5'-ATGCTAAGCGCCTTGTCTGT-3'

Supplementary Table 2. Antibodies information

Antibodies	Company	Catalog#	Application/Dilution
Anti-CHC	BD Transduction Laboratories	610499	WB (1:1000)
Anti-Asbt	Proteintech	20543-1-AP	WB (1:1000)
Anti-His_6	MBL life science	D291-3	WB (1:1000)
Anti-Myc	Proteintech	16286-1-AP	WB (1:1000)<br>IF (1:500)
Anti-FLAG	Proteintech	20543-1-AP	WB (1:1000)<br>IF (1:500)
Anti-Rab11	Proteintech	15903-1-AP	IHC (1:200)
Anti-ADRP/Perilipin 2	Proteintech	15294-1-AP	IHC (1:200)
anti-Villin	Proteintech	16488-1-AP	IHC (1:200)
Peroxidase affiniPure goat anti-mouse IgG secondary antibody	Jackson ImmunoResearch Laboratories	115-035-003	WB (1:5000)
Peroxidase AffiniPure goat anti-rabbit IgG secondary antibody	Jackson ImmunoResearch Laboratories	111-035-144	WB (1:5000)
Alexa Fluor 488 goat anti-rabbit IgG	Invitrogen	A11008	IF (1:500)
Alexa Fluor 555 goat anti-rabbit IgG	Invitrogen	A21422	IF (1:500)
