# Supplementary material for: Bile acids-mediated intracellular cholesterol transport promotes intestinal cholesterol absorption and NPC1L1 recycling
Source: Nat Commun. 2023 Oct 13;14:6469. doi: 10.1038/s41467-023-42179-5 (PMC10575946; doi:10.1038/s41467-023-42179-5)
Supplement: Supplementary file 3 — Reporting Summary [file 41467_2023_42179_MOESM3_ESM.pdf]

nature portfolio

Corresponding author(s): Bao-Liang Song

Last updated by author(s): Sep 14, 2023

Reporting Summary

Nature Portfolio wishes to improve the reproducibility of the work that we publish. This form provides structure for consistency and transparency in reporting. For further information on Nature Portfolio policies, see our Editorial Policies and the Editorial Policy Checklist.

Statistics

For all statistical analyses, confirm that the following items are present in the figure legend, table legend, main text, or Methods section.

n/a	Confirmed	
☐	☒	The exact sample size (n) for each experimental group/condition, given as a discrete number and unit of measurement
☐	☒	A statement on whether measurements were taken from distinct samples or whether the same sample was measured repeatedly
☐	☒	The statistical test(s) used AND whether they are one- or two-sided<br>*Only common tests should be described solely by name; describe more complex techniques in the Methods section.*
☒	☐	A description of all covariates tested
☐	☒	A description of any assumptions or corrections, such as tests of normality and adjustment for multiple comparisons
☐	☒	A full description of the statistical parameters including central tendency (e.g. means) or other basic estimates (e.g. regression coefficient) AND variation (e.g. standard deviation) or associated estimates of uncertainty (e.g. confidence intervals)
☐	☒	For null hypothesis testing, the test statistic (e.g. F, t, r) with confidence intervals, effect sizes, degrees of freedom and P value noted<br>*Give P values as exact values whenever suitable.*
☒	☐	For Bayesian analysis, information on the choice of priors and Markov chain Monte Carlo settings
☒	☐	For hierarchical and complex designs, identification of the appropriate level for tests and full reporting of outcomes
☒	☐	Estimates of effect sizes (e.g. Cohen's d, Pearson's r), indicating how they were calculated

Our web collection on statistics for biologists contains articles on many of the points above.

Software and code

Policy information about availability of computer code

Data collection	Bio-Rad CFX Connect Real-Time System; Microplate Manager 6 Software (Version 6.3); Tanon Imager 5200 (Version 2.03); CytExpert (Version 2.4.0.28); FlowJo (Version 10.0.7); Leica LAS X (Cersion 3.5.2.18963); Zetasizer Nano ZSP.
Data analysis	Confocal images were analyzed using Image J (Version 1.50i). Quantitative PCR were analyzed by CFX Manager (Version 3.1). Statistical analyses were performed using GraphPad Prism (Version 9.0). Data from flow cytometry was analyzed by FlowJo (Version 10.0.7).

For manuscripts utilizing custom algorithms or software that are central to the research but not yet described in published literature, software must be made available to editors and reviewers. We strongly encourage code deposition in a community repository (e.g. GitHub). See the Nature Portfolio guidelines for submitting code & software for further information.

Data

Policy information about availability of data

All manuscripts must include a data availability statement. This statement should provide the following information, where applicable:

- Accession codes, unique identifiers, or web links for publicly available datasets
- A description of any restrictions on data availability
- For clinical datasets or third party data, please ensure that the statement adheres to our policy

A Reporting Summary for this article describing our experiment details is available as Supplementary Information file. The data supporting the findings of this study are available within the paper and its supplementary information. The source data underlying Figures and Supplementary Figures are provided as a Source Data file. Specific data P values are also included within the Source Data file. Source data are provided with this paper.

Research involving human participants, their data, or biological material

Policy information about studies with human participants or human data. See also policy information about sex, gender (identity/presentation), and sexual orientation and race, ethnicity and racism.

Reporting on sex and gender	N/A
Reporting on race, ethnicity, or other socially relevant groupings	N/A
Population characteristics	N/A
Recruitment	N/A
Ethics oversight	N/A

Note that full information on the approval of the study protocol must also be provided in the manuscript.

Field-specific reporting

Please select the one below that is the best fit for your research. If you are not sure, read the appropriate sections before making your selection.

☒ Life sciences ☐ Behavioural & social sciences ☐ Ecological, evolutionary & environmental sciences

For a reference copy of the document with all sections, see nature.com/documents/nr-reporting-summary-flat.pdf

Life sciences study design

All studies must disclose on these points even when the disclosure is negative.

Sample size	For animal experiments, 4 mice were used in per group. For confocal images quantification, 300 cells from 3 independent trials were counted. For Flow cytometry analysis, 6000 cells from 3 independent trials were analyzed. For qPCR analysis or other analysis, 3 independent trials were performed. The sample size was chosen based on the suggestions from the reviewers and previous studies in the field.
Data exclusions	No data was excluded.
Replication	Numbers of replicates were stated in the figure legends.
Randomization	All cells and mice were randomly allocated to experimental groups.
Blinding	Single-blinding. Two different investigators were involved as follows: One investigator grouped the animals, treated the cells and conducted the experimental operation, and the other collected experimental data and statistical analysis.

Reporting for specific materials, systems and methods

We require information from authors about some types of materials, experimental systems and methods used in many studies. Here, indicate whether each material, system or method listed is relevant to your study. If you are not sure if a list item applies to your research, read the appropriate section before selecting a response.

Materials & experimental systems

n/a	Involved in the study
	☒ Antibodies
	☒ Eukaryotic cell lines
☒	☐ Palaeontology and archaeology
	☒ Animals and other organisms
☒	☐ Clinical data
☒	☐ Dual use research of concern
☒	☐ Plants

Methods

n/a	Involved in the study
☒	☐ ChIP-seq
	☒ Flow cytometry
☒	☐ MRI-based neuroimaging

Antibodies

Antibodies used

anti-CHC antibody (BD Transduction Laboratories, Cat# 610499, Lot# 610500, 1:1000 for immunoblotting);
anti-Asbt antibody (Proteintech, Cat# 20543-1-AP, Lot# 00076145, 1:1,000 for immunoblotting);
anti-His6 antibody (MBL life science, Cat# D291-3, Lot# 010, 1:1000 for immunoblotting);
anti-Myc antibody (Proteintech, Cat# 16286-1-AP, Lot# 00096544, 1:500 for immunofluorescent staining and 1:1,000 for immunoblotting);
anti-FLAG antibody (Proteintech, Cat# 20543-1-AP, Lot# 00089080, 1:1,000 for immunoblotting);
anti-Rab11 antibody (Proteintech, Cat# 15903-1-AP, Lot# 00091672, 1:200 for immunohistochemistry);
anti-ADRP/Perilipin 2 antibody (Proteintech, Cat# 15294-1-AP, Lot# 00114919, 1:200 for immunohistochemistry);
anti-Villin antibody (Proteintech, Cat# 16488-1-AP , Lot# 00047067, 1:200 for immunohistochemistry);
Peroxidase affiniPure goat anti-mouse IgG secondary antibody (Jackson ImmunoResearch Laboratories, Cat# 115-035-003, Lot# 146022, 1:5000);
peroxidase AffiniPure goat anti-rabbit IgG secondary antibody (Jackson ImmunoResearch Laboratories, Cat# 111-035-144, Lot# UH283636, 1:5000);
Alexa Fluor 488 goat anti-rabbit IgG (Invitrogen, Cat# A11008, Lot# 2256822, 1:500 for immunofluorescence);
Alexa Fluor 555 goat anti-rabbit IgG (Invitrogen, Cat# A21422, Lot# 2339822, 1:500 for immunofluorescence);
Anti-NPC1L1 (1:200 for immunohistochemistry (about 5 μg/mL) and 1:1,000 for immunoblotting (about 1 μg/mL)) was homemade by rabbit immunization in our laboratory;
Anti-SREBP2 Antibody (1:100, about 5 μg/mL) was produced and purified from hybridoma cell line 1D2 (ATCC, Cat Num #CRL-2545) in our laboratory.

Validation

anti-CHC antibody (BD Transduction Laboratories, #610499);
https://www.bdbiosciences.com/en-eu/products/reagents/microscopy-imaging-reagents/immunofluorescence-reagents/purified-mouse-anti-clathrin-heavy-chain.610499

anti-Asbt antibody (Proteintech, Cat# 20543-1-AP);
https://www.ptgcn.com/products/ASBT-Antibody-25245-1-AP.htm

anti-His6 antibody (MBL life science, Cat# D291-3);
https://ruo.mbl.co.jp/bio/e/dtl/A/?pcd=D291-3

anti-Myc antibody (Proteintech, Cat# 16286-1-AP);
https://www.ptgcn.com/products/MYC-tag-Antibody-16286-1-AP.htm

anti-FLAG antibody (Proteintech, Cat# 20543-1-AP);
https://www.ptgcn.com/products/Flag-Tag-Antibody-20543-1-AP.htm#product-information

anti-Rab11 antibody (Proteintech, Cat# 15903-1-AP);
https://www.ptgcn.com/products/RAB11A-Antibody-15903-1-AP.htm

anti-ADRP/Perilipin 2 antibody (Proteintech, Cat# 15294-1-AP);
https://www.ptgcn.com/products/ADRP-Antibody-15294-1-AP.htm

anti-Villin antibody (Proteintech, Cat# 16488-1-AP);
https://www.ptgcn.com/products/VIL1-Antibody-16488-1-AP.htm

Peroxidase affiniPure goat anti-mouse IgG secondary antibody (Jackson ImmunoResearch Laboratories, Cat# 115-035-003);
https://www.jacksonimmuno.com/catalog/products/115-035-003

Peroxidase AffiniPure goat anti-rabbit IgG secondary antibody (Jackson ImmunoResearch Laboratories , Cat# 111-035-144);
https://www.jacksonimmuno.com/catalog/products/111-035-144

Alexa Fluor 488 goat anti-rabbit IgG (Invitrogen, Cat# A11008);
https://www.thermofisher.cn/cn/zh/antibody/product/Goat-anti-Rabbit-IgG-H-L-Cross-Adsorbed-Secondary-Antibody-Polyclonal/

A-11008

Alexa Fluor 555 goat anti-rabbit IgG (Invitrogen, Cat# A21422); https://www.thermofisher.cn/cn/zh/antibody/product/Goat-anti-Mouse-IgG-H-L-Cross-Adsorbed-Secondary-Antibody-Polyclonal/A-21422

Anti-NPC1L1 antibody (ref. 1) and mouse monoclonal anti-SREBP2 (1D2) antibody (ref. 2) were validated in our previous work.

Ref 1: Xie, C., et al., Ezetimibe blocks the internalization of NPC1L1 and cholesterol in mouse small intestine. Journal of Lipid Research, 2012. 53(10): p. 2092-2101.

Ref 2: Xiao, J., et al., POST1/C12ORF49 regulates the SREBP pathway by promoting site-1 protease maturation. Protein & Cell, 2021. 12(4): p. 279-296.

Eukaryotic cell lines

Policy information about cell lines and Sex and Gender in Research

Cell line source(s)	CRL1601 cells (Female) were purchased from ATCC (Cat# CRL1601). CRL1601/NPC1L1-3×Myc-EGFP cells were generated by lentiviral infection and single-cell cloning in our previous work (Ref 3). CRL1601/NPC1L1-3×Myc-EGFP-ΔA1272LAL (CRL1601 cells stably expressing NPC1L1-3×Myc-EGFP with a deletion of ΔA1272LAL) and CRL1601/NPC1L1-3×Myc-EGFP-A1272LAL→4E (CRL1601 cells stably expressing NPC1L1-3×Myc-EGFP with A1272LAL-to-E1272EEE substitution) stable cell lines were generated by lentiviral infection and single-cell cloning in this study.<br><br>Ref 3: Ge, L., et al., Flotillins play an essential role in Niemann-Pick C1-like 1-mediated cholesterol uptake. Proceedings of the National Academy of Sciences, 2011. 108(2): p. 551-556.
Authentication	No further authentication of the cell lines was performed before use.
Mycoplasma contamination	Cell lines were not tested for mycoplasma.
Commonly misidentified lines (See ICLAC register)	No commonly misidentified cell lines were used in this study.

Animals and other research organisms

Policy information about studies involving animals; ARRIVE guidelines recommended for reporting animal research, and Sex and Gender in Research

Laboratory animals	Male C57BL/6J mice (9 and 12 weeks of age) were purchased from Centers for Disease Control (Hubei, China). The Npc1l1-EGFP knock-in C57BL/6N mouse was a kind gift from Prof. Weiping Zhang at Naval Medical University in China. The Npc1l1-Y1306VNxxF→AAAxxA knock-in mouse was generated from C57BL/6JGpt by CRISPR-cas9 system (GemPharmatech). Neonatal NPC1L1-EGFP knock-in C57BL/6N male mice was used for in vitro intestinal culture assay and 12-week-old male knock-in mice were applied to bile duct cannulation assay. Littermates from the heterozygous of Npc1l1-Y1306VNxxF→AAAxxA crossing were wild-type controls. Mice was housed in plastic cages in a specific pathogen-free animal facility under a 12-hour day/light cycle at 22 °C with a humidity of 50–60%. Mice were fed on chow diet (Beijing HFK Biosciences, 1026) ad libitum.
Wild animals	The study did not involve wild animals.
Reporting on sex	No gender-based analyses have been performed in this study. Male mice were chosen because they are widely used in the field.
Field-collected samples	The study did not involve field-collected samples.
Ethics oversight	All animal experiments were performed according to the protocols (WDSKY0201408) approved by the Institutional Animal Care and Use Committee of Wuhan University.

Note that full information on the approval of the study protocol must also be provided in the manuscript.

Flow Cytometry

Plots

Confirm that:

- [x] The axis labels state the marker and fluorochrome used (e.g. CD4-FITC).
- [x] The axis scales are clearly visible. Include numbers along axes only for bottom left plot of group (a 'group' is an analysis of identical markers).
- [x] All plots are contour plots with outliers or pseudocolor plots.
- [x] A numerical value for number of cells or percentage (with statistics) is provided.

Methodology

Sample preparation	CRL1601/NPC1L1-3×Myc-EGFP cells were washed with 1×PBS for 2 times, suspended using 2 mM EDTA in 1×PBS and then rinsed with ice-cold 1×PBS for once. CRL1601/NPC1L1-3×Myc-EGFP cells were fixed with 4% PFA for 30 min at 4 °C. Next, cells were incubated with anti-Myc antibody and secondary antibody (diluted in with 1% w/v BSA) at 4 °C for 1 h in turn. Samples were washed with 1×PBS for 3 times, resuspended with 1% BSA in 1×PBS and then analyzed by CytoFLEX.
Instrument	Beckman Coulter CytoFLEX.
Software	CytExpert (Version 2.4.0.28); FlowJo (Version 10.0.7).
Cell population abundance	6000 cells (2000 cells/trial) from 3 indenpendent trials were analyzed in each experiment.
Gating strategy	See Figure S11. Briefly, the normal sized stable cells (CRL1601/NPC1L1-3×Myc-EGFP cells, CRL1601/NPC1L1-3×Myc-EGFP-ΔA1272LAL or CRL1601/NPC1L1-3×Myc-EGFP-A1272LAL→4E) were gated using forward scatter and side scatter. Then single cells from the stable cells were gated by forward scatter height and area, and used for fluorescence intensity measurement.

☒ Tick this box to confirm that a figure exemplifying the gating strategy is provided in the Supplementary Information.
